# Marliolide Derivative Induces Melanosome Degradation via Nrf2/p62-Mediated Autophagy

**DOI:** 10.3390/ijms22083995

**Published:** 2021-04-13

**Authors:** Cheong-Yong Yun, Nahyun Choi, Jae Un Lee, Eun Jung Lee, Ji Young Kim, Won Jun Choi, Sang Ho Oh, Jong-Hyuk Sung

**Affiliations:** 1 STEMORE Co. Ltd., Incheon 21983, Korea; cyyun@stemore.co.kr (C.-Y.Y.); nh147837@gmail.com (N.C.); 2 College of Pharmacy, Dongguk University-Seoul, Goyang 10326, Gyeonggi-do, Korea; ljwoon55@naver.com (J.U.L.); mp89@dongguk.edu (W.J.C.); 3 Department of Dermatology and Cutaneous Biology Research Institute, Severance Hospital, Yonsei University College of Medicine, Seoul 03722, Korea; leeej87@yuhs.ac (E.J.L.); snyd@yuhs.ac (J.Y.K.); 4 Brain Korea 21 PLUS Project for Medical Science, Yonsei University College of Medicine, Seoul 03722, Korea; 5 Institute of Pharmaceutical Sciences, College of Pharmacy, Yonsei University, Incheon 21983, Korea

**Keywords:** Nrf2, p62, autophagy, melanosome degradation

## Abstract

Nuclear factor erythroid 2-related factor 2 (Nrf2), which is linked to autophagy regulation and melanogenesis regulation, is activated by marliolide. In this study, we investigated the effect of a marliolide derivative on melanosome degradation through the autophagy pathway. The effect of the marliolide derivative on melanosome degradation was investigated in α-melanocyte stimulating hormone (α-MSH)-treated melanocytes, melanosome-incorporated keratinocyte, and ultraviolet (UV)B-exposed HRM-2 mice (melanin-possessing hairless mice). The marliolide derivative, 5-methyl-3-tetradecylidene-dihydro-furan-2-one (DMF02), decreased melanin pigmentation by melanosome degradation in α-MSH-treated melanocytes and melanosome-incorporated keratinocytes, evidenced by premelanosome protein (PMEL) expression, but did not affect melanogenesis-associated proteins. The UVB-induced hyperpigmentation in HRM-2 mice was also reduced by a topical application of DMF02. DMF02 activated Nrf2 and induced autophagy in vivo, evidenced by decreased PMEL in microtubule-associated proteins 1A/1B light chain 3B (LC3)-II-expressed areas. DMF02 also induced melanosome degradation via autophagy in vitro, and DMF02-induced melanosome degradation was recovered by chloroquine (CQ), which is a lysosomal inhibitor. In addition, Nrf2 silencing by siRNA attenuated the DMF02-induced melanosome degradation via the suppression of p62. DMF02 induced melanosome degradation in melanocytes and keratinocytes by regulating autophagy via Nrf2-p62 activation. Therefore, Nrf2 activator could be a promising therapeutic agent for reducing hyperpigmentation.

## 1. Introduction

Melanin pigmentation is a complex process that starts the oxidation of tyrosine to its metabolite, L-3,4-Dihydroxyphenylalanine (L-DOPA), by tyrosinase (TYRO), a rate-limiting enzyme [1,2]. Melanogenesis is influenced by extrinsic and intrinsic factors, such as hormones, inflammation, and ultraviolet (UV) exposure [3,4]. Melanin produced by melanocytes is known to protect the skin from UV radiation [5]. However, an excessive accumulation of melanin in the skin results in hyperpigmentary disorders or cosmetic concerns, such as melasma or freckles [6]. Therefore, there are tremendous efforts to reduce melanin production in cosmetics and pharmaceutical industries.

Autophagy is the natural process that removes unnecessary or dysfunctional components in cells [7,8]. In melanocytes, autophagy is involved in the opposite functions of melanosome biogenesis [9] and degradation [10]. For example, microtubule-associated proteins 1A/1B light chain 3B (LC3), a marker protein of autophagy, contributes to melanogenesis by increasing ERK-dependent microphthalmia-associated transcription factor (MITF) expression [9]. However, other data revealed that autophagy had a pivotal role in skin color determination by regulating melanosome degradation in keratinocytes [10]. Suppression of autophagy dysregulated the antioxidant response and resulted in premature senescence of melanocytes [11]. In addition, autophagy deficient melanocytes showed a senescence associated secretory phenotype, and autophagy deficient melanocytes or keratinocytes displayed only moderate reduction of pigmentation [12].

The nuclear factor E2-related factor 2 (Nrf2) is a transcription factor responsible for inducing antioxidant enzymes [13,14]. Under quiescent conditions, Nrf2 was constitutively degraded through the ubiquitin-proteasome pathway [15]. Under oxidative stress, Nrf2 is released from the Nrf2-kelch-like ECH-associated protein 1 (Keap1) complex and transferred to the nucleus, where it activates phase II antioxidant enzymes, such as heme oxygenase-1 (HO-1) and NADH quinine oxidoreductase 1 (Nqo1) [16]. PI3K-AKT signaling via Nrf2 protects against hyperoxia-induced acute lung injury, promoting inflammation [17,18]. Nrf2 signaling pathway plays a critical role in protecting melanocytes from oxidative damage [19]. The dysfunction of the Nrf2 signaling pathway increased the sensitivity of vitiligo melanocytes to H_2_O_2_-induced oxidative damage [20]. Dietary phenolics and loliolide inhibited UVA-induced melanogenesis through Nrf2-dependent antioxidant responses [21,22]. Of interest, overexpression of Nrf2 decreased the expression of TYRO and tyrosinase-related protein 1 [23]. Although the inhibitory effects of Nrf2 on melanin synthesis have been reported, its underlying molecular mechanism is not yet clearly identified.

Marliolide, which activates a transcription factor, Nrf2, is a natural product extracted from *Mollinedia* leaves and *Cinnamomum cassia* barks [24]. Marliolide inhibited the formation of the Nrf2-Keap1 complex by binding to Keap1, thereby it led to suppression of Nrf2 degradation [24]. Marliolide promoted the expression of antioxidant enzymes to protect the cells from oxidative stress [5,24] and increased p62 expression to modulate autophagy [25]. However, the whitening effect of marliolide has not been previously reported. Therefore, we first investigated whether marliolide and its derivatives can induce melanin degradation, and DMF02 was chosen as the best whitening compound via Nrf2-p62 axis activation. We further studied the underlying molecular mechanism of DMF02 for hypopigmentation effect.

## 2. Results

### 2.1. Marliolide or Marliolide Derivatives Reduced Melanin Products in B16F0 Melanoma Cells

**Figure 1:**
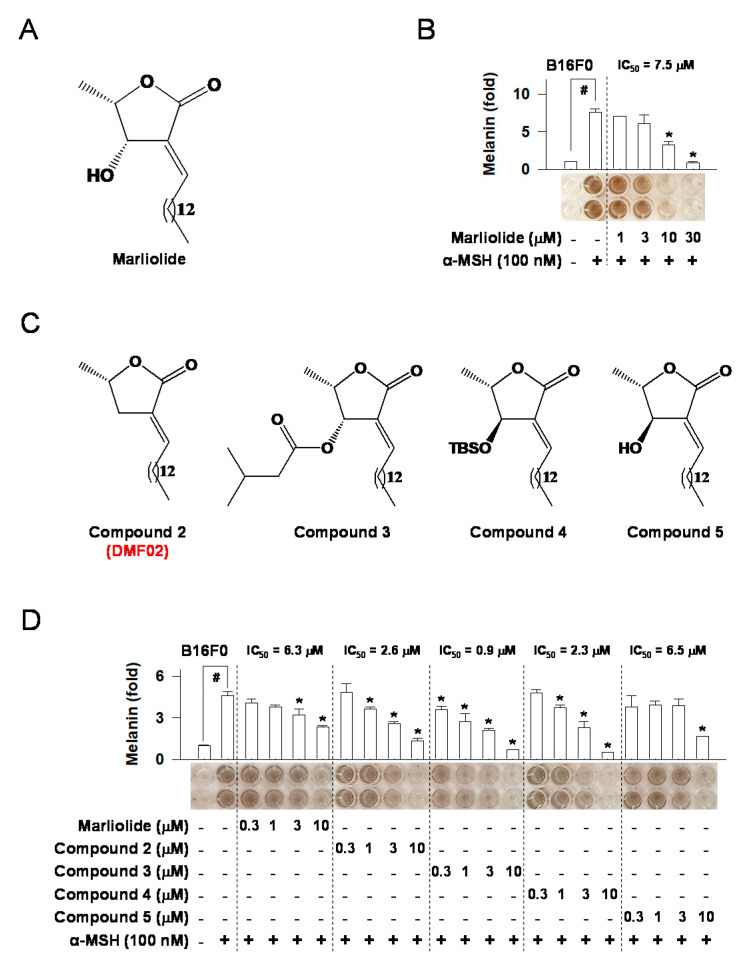
The effect of marliolide or marliolide derivatives on melanin products in B16F0 melanoma cells. The chemical structure of marliolide (**A**) or marliolide derivatives (**C**). B16F0 melanoma cells were stimulated with α-MSH for 72 h in the presence of marliolide (**B**) or marliolide derivatives (**D**). All values are expressed as mean ± SEM from two independent experiments in duplicate. # *p* < 0.05 vs. medium only group. * *p* < 0.05 vs. α-MSH-stimulated group. The difference was determined using the Student’s *t*-test.

As marliolide (Figure 1A) was reported to increase antioxidant activity by promoting the expression of antioxidant enzymes, such as HO-1 and Nqo1 [16], we first investigated its hypopigmentation effects on B16F0 melanoma cells. Marliolide, at the concentrations of 1 to 30 μM, did not affect cell viability (Figure S1A). Additionally, it reduced α-MSH-induced melanin production in a dose-dependent manner (Figure 1B). After confirming the hypopigmentation effect of marliolide, we synthesized four marliolide derivatives (compounds 2–5) to improve pharmacokinetics profiles with increased stability and better cellular penetration (Figure 1C). Marliolide and its derivatives, at 0.3–30 μM, had no impact on cell viability (Figure S1B). Like marliolide, all derivatives also reduced α-MSH-induced melanin production in a dose-dependent manner (Figure 1D). Eventually, compound 2 (5-methyl-3-tetradecylidene-dihydro-furan-2-one, DMF02) was selected for further study to discover underlying mechanisms of the hypopigmentation effect, considering the advantages of compound 2, such as easy synthesis.

### 2.2. DMF02 Reduced UVB-Induced Pigmentation in HRM-2 Mice Skin

**Figure 2:**
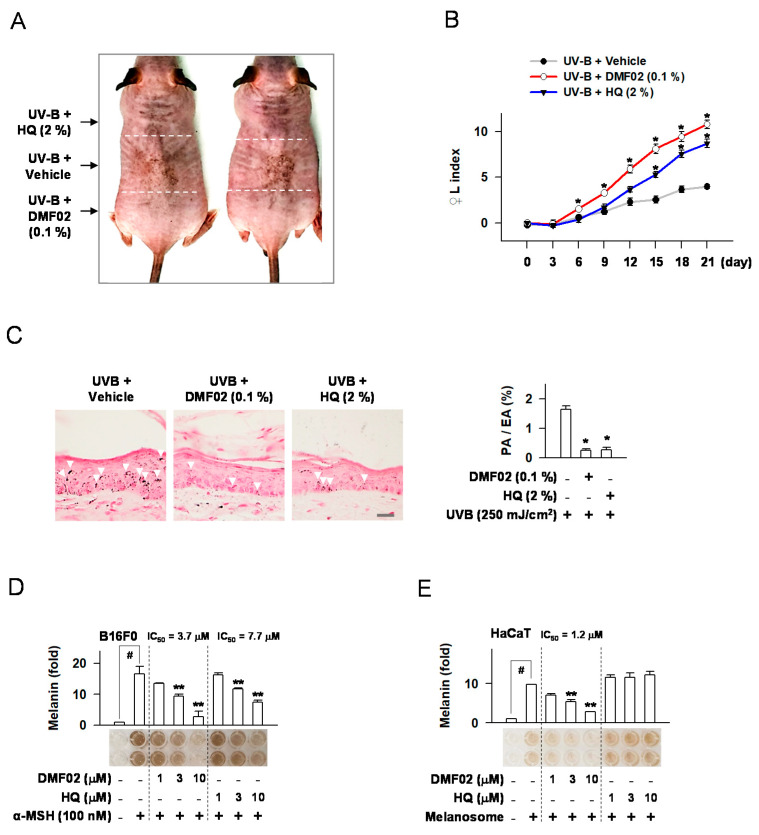
Effects of DMF02 on melanin pigmentation in vivo and in vitro. (**A**) Image of skin pigmentation in HRM2 mice. Black arrows showing the allocation of the dorsal skin of mice. (**B**) The change of lightening index (△L) in UVB-exposed skin. (**C**) UVB-exposed skin that was stained with Fontana-Masson silver nitrite. White arrows indicate the visible pigmented area in skin tissue, and relative pigmentation change (pigmented area/epidermal area (PA/EA) ratio) are presented. Scale bar 20 μm. B16F0 melanoma (**D**) or HaCaT cells (**E**) were treated with α-MSH or isolated melanosomes for 78 h in the presence of DMF02. The amount of melanin was represented as a relative fold. All values are expressed as mean ± SEM from two independent experiments in duplicate. * *p* < 0.05 vs. UVB plus vehicle alone. # *p* < 0.05 vs. medium alone-added group. ** *p* < 0.05 vs. α-MSH or melanosome-treated group, Student’s *t*-test.

HRM-2 mice, a melanin-possessing hairless breed, were used to evaluate DMF02’s hypopigmentation effects. After inducing skin pigmentation in the mice by exposure to UVB light (150–250 mJ/cm^2^), topical treatment of 0.1% DMF02 or 2% hydroquinone (HQ) was applied for 21 days (Figure S2). DMF02 and HQ’s effect in reducing melanin pigmentation was examined by measuring the darkness of DMF02 and HQ-treated areas compared to vehicle-treated areas (Figure 2A). The increase in the lightening index (△L index) was significantly greater in DMF02 or HQ-treated areas than that of the vehicle-treated areas (*p* = 0.00004 and 0.006, respectively) (Figure 2B). Next, melanin pigmentation was quantified in Fontana-Masson stained mouse skin tissues. The DMF02 or HQ-treated areas displayed significantly reduced melanin pigmentation in skin tissues compared to vehicle-treated areas (*p* < 0.05 in both) (Figure 2C). The results indicate that DMF02, a Nrf2 activator, reduces UVB induced hyperpigmentation in HRM-2 mice skin.

### 2.3. DMF02 Decreased Melanin Production in Melanocytes and Keratinocytes Containing Transferred Melanosomes

First, α-MSH or other cyclic adenosine monophosphate (cAMP) activators, such as N^6^,2’-O-dibutyryl (db)-cAMP and forskolin, were used to induce melanin production in melanocytes, such as B16F0, MNT1, and human epidermal melanocytes (HEM). DMF02 or HQ was then incubated with α-MSH or other cAMP activators. Both DMF02 and HQ treatment reduced α-MSH or other cAMP activators-induced melanin production in a dose-dependent manner (Figure 2D, Figures S3A,B and S4A,B). Next, the effect of DMF02 and HQ on keratinocytes containing transferred melanosomes were evaluated. Isolated melanosomes from MNT1 cells were added to the culture media of keratinocytes, such as HaCaT and human epidermal keratinocytes (HEK) cells, and isolated melanosomes were transferred into the cytosol of the keratinocytes. DMF02 treatment was observed to decrease intracellular melanosomes in keratinocytes in a dose-dependent manner, while HQ did not affect intracellular melanosomes (Figure 2E and Figure S3C). Our results showed that DMF02 decreased the melanin pigmentation in melanocytes and keratinocytes. In contrast, HQ, a TYRO inhibitor, inhibited melanin production only in melanocytes. These data suggested that HQ inhibited melanin production in melanocytes, while DMF02 may induce melanin degradation in both melanocytes and keratinocytes.

### 2.4. DMF02 Decreased Melanosome in Melanocytes and Keratinocytes

**Figure 3:**
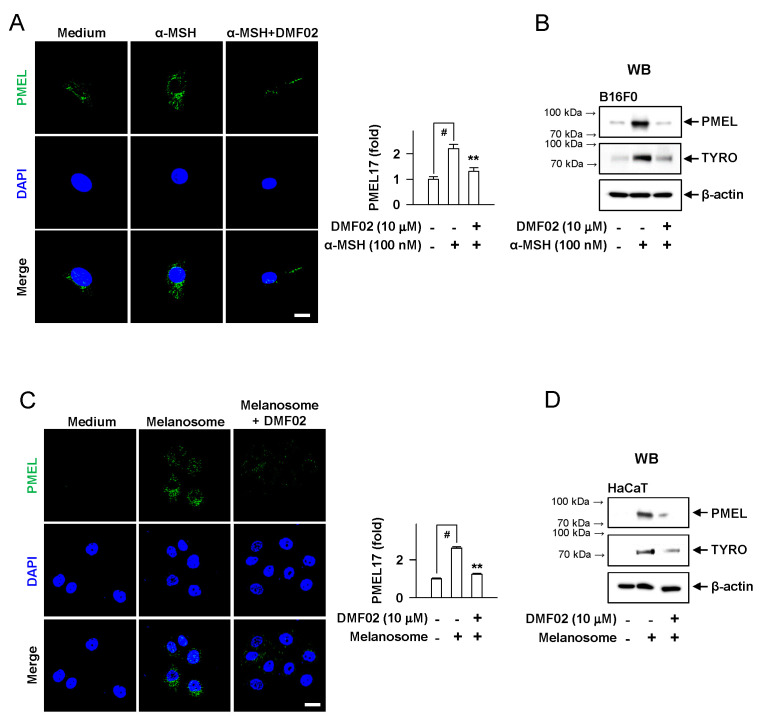
Effects of DMF02 on melanosome degradation. B16F0 melanoma cells (**A**) or HaCaT cells (**C**) were stained for premelanosome protein (PMEL). Intensity of PMEL was represented as a relative fold. B16F0 melanoma cells (**B**) or HaCaT cells (**D**) were treated with α-MSH or isolated melanosomes in the absence or presence of DMF02. Scale bar 20 μm. Data are mean ± SEM. *p* < 0.05 vs. UVB plus vehicle alone. # *p* < 0.05 vs. medium alone-added group. ** *p* < 0.05 vs. α-MSH-stimulated group or melanosome-treated group. The difference was determined using the Student’s *t*-test.

In B16F0 melanoma cells, DMF02 attenuated the increase in PMEL and TYRO protein levels, following α-MSH-induced melanogenesis (Figure 3A,B). Additionally, the melanosomes incorporated into HaCaT keratinocytes were significantly decreased by DMF02 treatment (Figure 3C,D). However, DMF02 did not affect the mRNA and protein levels of MITF and mRNA of TYRO in the B16F0 cell line, which were induced by α-MSH (Figure S5A–C). The incubation of arbutin, a TYRO inhibitor, with L-DOPA and TYRO in cell-free media significantly decreased L-DOPA oxidation; in the same condition, DMF02 did not affect L-DOPA oxidation (Figure S5D). These data suggested that the hypopigmentation effect of DMF02 was attributable to melanosome degradation in melanocytes and keratinocytes, but not to inhibition of melanogenesis or TYRO expression.

### 2.5. DMF02 Increased Autophagy in HRM-2 Mice Skin

**Figure 4:**
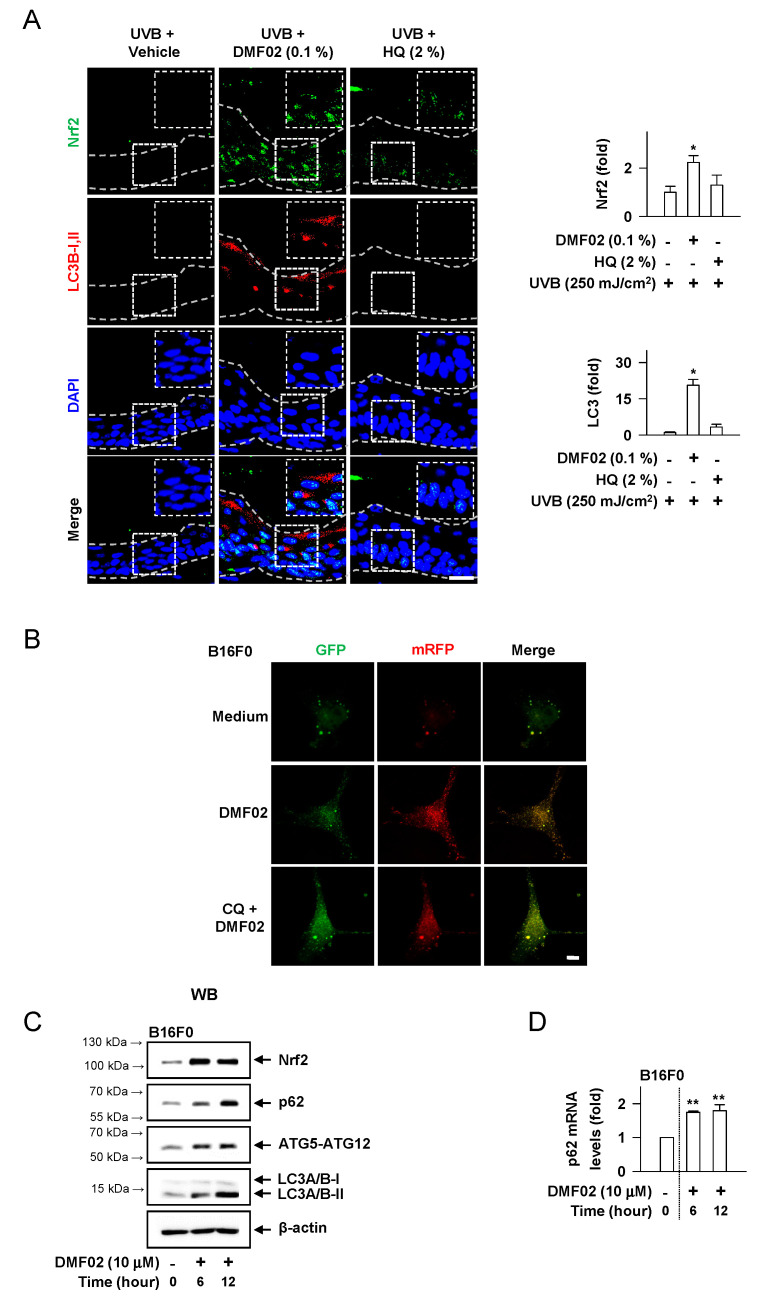

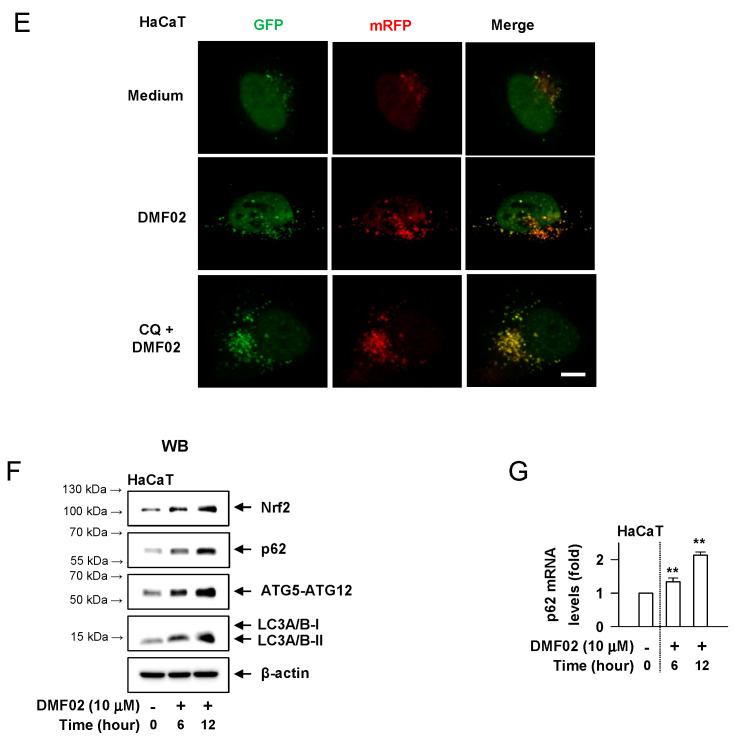
Effects of DMF02 on autophagy. (**A**) The dorsal skin of HRM2 mice was co-stained for Nrf2 (green) and LC3B-I, II (red). Intensity of Nrf2 or LC3B-I, II was represented as a relative fold. Scale bar 20 μm. B16F0 melanoma cells (**B**) and HaCaT cells (**E**) transfected with RFP-GFP-LC3 plasmid was treated with DMF02 (10 μM) for 8 h and 1 hr preincubation with chloroquine (CQ) and then subjected to confocal microscopy to evaluate autophagic flux. Yellow puncta represent autophagosomes and red puncta represent autolysosomes. Scale bar 10 μm. Western blotting (WB) of Nrf2, p62, ATG5-ATG12 complex, and LC3A/B-I, II. B16F0 melanoma cells (**C**) or HaCaT cells (**F**) were treated with DMF02 for the indicated time. Real-time PCR on the induction of p62. B16F0 melanoma cells (**D**) or HaCaT cells (**G**) were treated with DMF02 for indicated time. Data are mean ± SEM. * *p* < 0.05 vs. UVB plus vehicle alone. ** *p* < 0.05 vs. medium alone-added group. The difference was determined using the Student’s *t*-test.

We evaluated whether Nrf-p62 mediated autophagy was responsible for the melanosome degradation caused by DMF02. The topical application of DMF02 enhanced the expression of Nrf2 and LC3-II, another autophagy marker, in HRM-2 mice skin, compared to vehicle and HQ-treated skin (Figure 4A). Confocal microscopy using cells transfected with mRFP-GFP-LC3 confirmed that the DMF02 treatment induced autophagic flux in B16F0 melanoma cells and HaCaT cells (Figure 4B,E). The number of yellow puncta (autophagosomes) in the cells were significantly increased after DMF02 treatment in the presence of chloroquine (CQ), which inhibits autophagosome-lysosome fusion, while the number of red puncta (autolysosomes) in the cells were significantly increased after DMF02 treatment in the absence of CQ, indicating that DMF02 increases autophagy activity. Next, the response of Nrf2 and autophagy markers, including p62, ATG5-ATG12 complex, and LC3, to DMF02 treatment were evaluated in B16F0 melanoma cells and HaCaT keratinocytes. The protein level of Nrf2 and autophagy markers, such as ATG5-ATG12 complex and LC3-II, were significantly increased in melanocytes and keratinocytes after 6 h and 12 h of DMF02 treatment; on the other hand, p62 protein and mRNA level were increased after DMF02 treatment (Figure 4C,D,F,G). Thus, DMF02 not only increased Nrf2 expression but also induced autophagy activation in melanocytes and keratinocytes.

### 2.6. DMF02 Reduced Melanin-Containing Melanosome via Autophagy in Melanocytes and Keratinocytes

**Figure 5:**
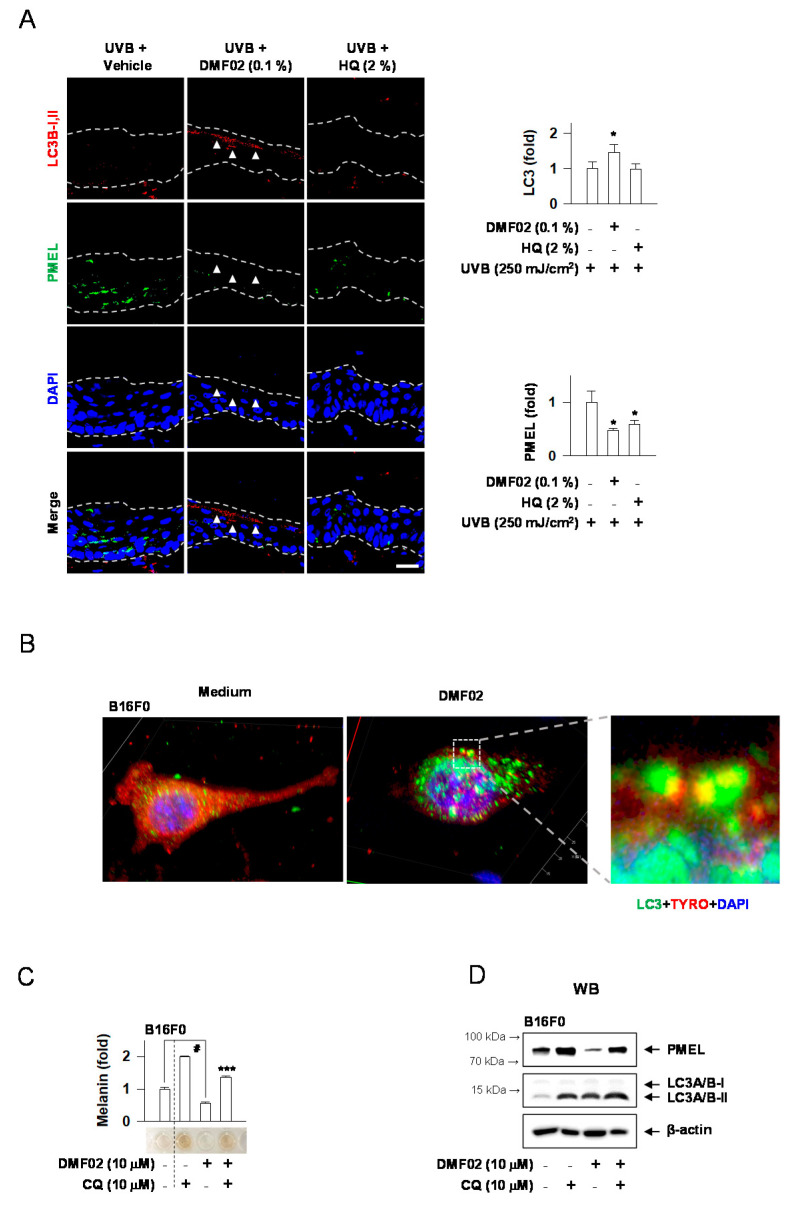

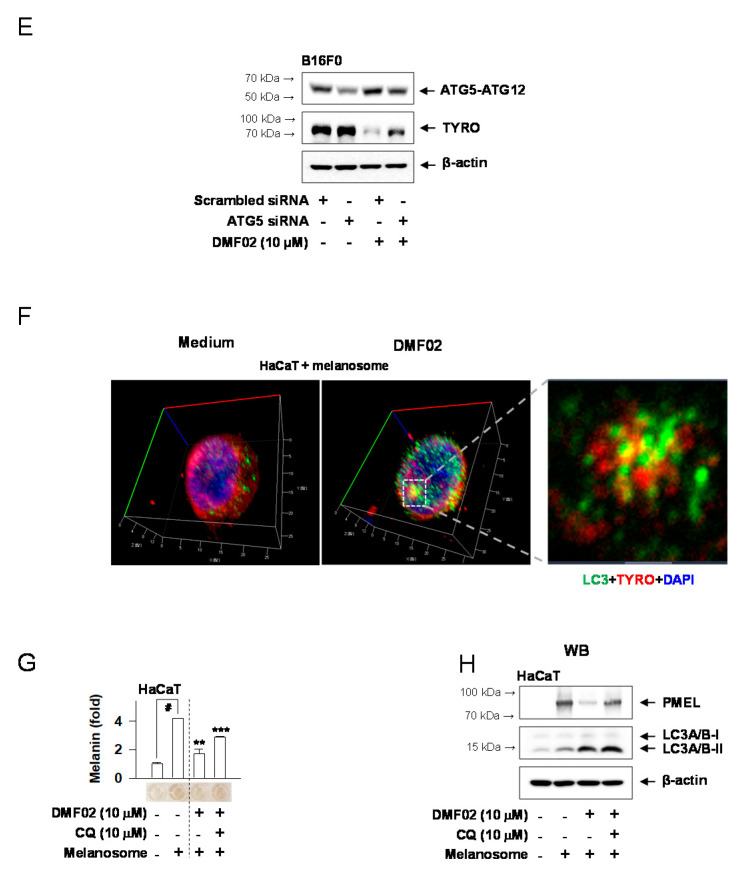
DMF02 reduced melanosome via autophagy. (**A**) The dorsal skin of HRM2 mice was co-stained for LC3B-I, II (red, white arrowhead) and PMEL (green). Confocal microscopy of B16F0 melanoma cells (**B**) and isolated melanosome-loaded HaCaT cells (**F**) treated with DMF02 for 12 h and colocalization of melanosomes (TYRO, red) and autophagosomes (LC3, green) were detected. B16F0 melanoma cells (**C**) or melanosome-incorporated HaCaT cells (**G**) were treated with DMF02 for 78 h in the presence of CQ. The amount of melanin was quantified by absorbance values, and measured at 405 nm. WB of PMEL. B16F0 melanoma cells (**D**) or melanosome-incorporated HaCaT cells (**H**) were treated with DMF02 for 48 h in the presence of CQ. (**E**) Expressions of TYRO were examined with western blotting after 1 day treatment with DMF02 following ATG5 silencing. Scale bar 20 μm. All values are expressed as mean ± SEM from two independent experiments in duplicate. * *p* < 0.05 vs. UVB plus vehicle alone. # *p* < 0.05 vs. medium alone-added group. ** *p* < 0.05 vs. melanosome-treated group. *** *p* < 0.05 vs. DMF02-treated group, Student’s *t*-test.

We performed immunostaining of LC3-II and PMEL, markers for autophagy and melanosome, respectively, in UVB-irradiated HRM-2 mice skin after DMF02 or HQ treatment. DMF02 and HQ-treated skins appeared to have significantly lower expression of PMEL than the vehicle-applied areas. However, only DMF02-treated areas showed a higher expression of LC3-II (Figure 5A). Interestingly, the loss of PMEL in the LC3-expressing epidermis was notable. Confocal microscopy showed that DMF02 treatment induced colocalized expression of TYRO (red) and LC3 (green) in B16F0 melanoma cells and melanosome-incorporated HaCaT cells (Figure 5B,F), suggesting the sequestration of melanosomes by autophagosomes. Thus, these data suggest that DMF02-induced autophagy is closely related to melanosome degradation in both melanocytes and keratinocytes. Next, we sought to observe whether DMF02 reduced melanosomes in melanocytes and keratinocytes via an autophagic mechanism in the presence of CQ. The melanin-containing melanosomes decreased in DMF02-treated B16F0 cells, while the same effect was mitigated by CQ (Figure 5C). Additionally, DMF02 treatment decreased melanosomes in HaCaT keratinocytes containing transferred melanosomes (Figure 5G). CQ is commonly used to measure autophagic flux through the monitoring of LC3 turnover. Therefore, we performed western blot assay for PMEL and LC3 to show that melanosome degradation might be induced by autophagic activity of DMF02. As shown in Figure 5D,H, a decrease in the levels of PMEL by DMF02 treatment was abrogated by the presence of CQ, along with increased conversion to LC3 II by CQ (Figure 5D,H). These data suggest that DMF02 could decrease melanin pigmentation by autophagy-induced melanosome degradation in melanocytes and keratinocytes. To prove whether DMF02-mediated melanosome degradation was done by autophagic induction, we performed ATG5 silencing using ATG5 siRNA. As shown in Figure 5E, decreased expression of TYRO caused by DMF02 were ablated in ATG5-knockdown B16F0 cell lines. 

### 2.7. Nrf2 Knockdown Decreased DMF02-Induced Autophagy, Blocking Melanosome Degradation in Melanocytes

**Figure 6:**
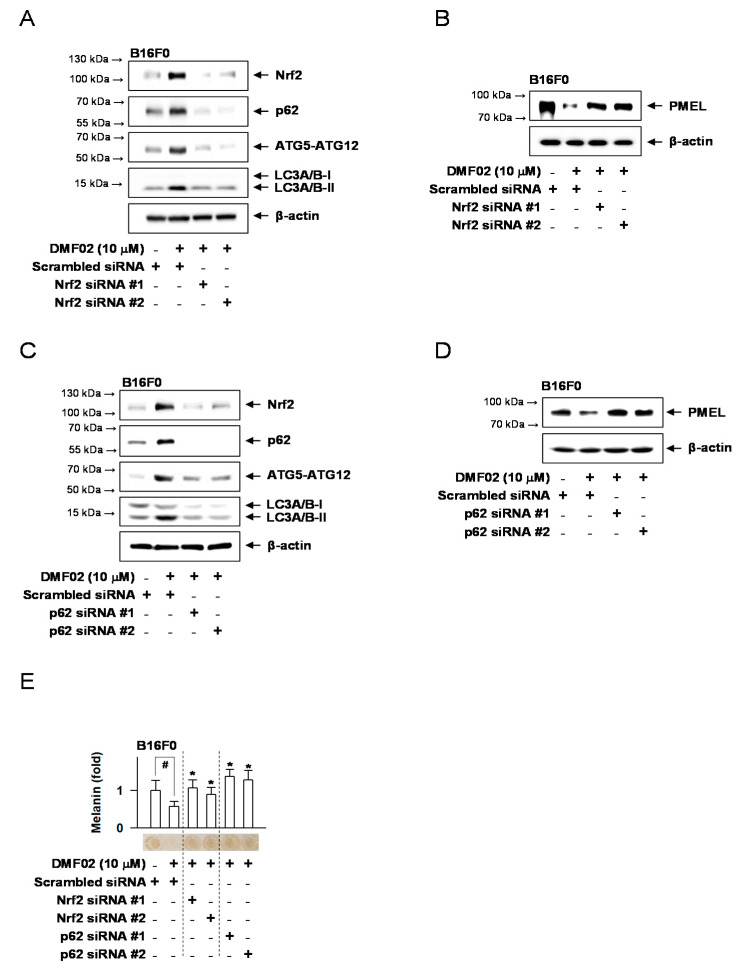
Nrf2 or p62 knockdown affects melanosome reduction via autophagy. WB of Nrf2, p62, ATG5-ATG12 complex, LC3A/B-I, II, or PMEL. B16F0 cells were transfected with siRNA against Nrf2 (**A**,**B**) or p62 (**C**,**D**) for 24 h and treated with DMF02 for 12 h or 48 h. (**E**) B16F0 cells were transfected with siRNA against Nrf2 or p62 for 24 h and treated with DMF02 for 78 h. The amount of melanin was quantified by absorbance values, measured at 405 nm, and are represented as a relative fold. All values are expressed as mean ± SEM from two independent experiments in duplicate. # *p* < 0.05 vs. scrambled siRNA plus medium alone-added group. * *p* < 0.05 vs. scrambled siRNA plus DMF02-treated group. The difference was determined using the Student’s *t*-test.

B16F0 melanoma cells with silenced Nrf2 or p62 were used to investigate whether DMF02-induced melanosome degradation was caused by Nrf2 and p62-mediated autophagy. DMF02-induced expression of Nrf2 and autophagic markers, including p62, ATG5-ATG12 complex, and LC3-II, were not observed in Nrf2-knockdown cells (Figure 6A). PMEL expression, which was decreased by DMF02 in Nrf2-expressing cells, recovered in Nrf2-silenced cells (Figure 6B). Similarly, DMF02 treatment did not increase ATG5-ATG12 complex and LC3-II expression in the p62 knockdown cells (Figure 6C). Additionally, the reduced expression of PMEL was not observed in the p62 knockdown cells despite DMF02 treatment (Figure 6D). Lastly, DMF02 treatment reduced melanin-containing melanosomes in cells treated with scrambled siRNA, whereas DMF02 treatment reduces melanosomes in the Nrf2 or p62 knockdown cells (Figure 6E). The results indicated that Nrf2 activation may regulate pigmentation through melanosome degradation via p62-mediated autophagy activation.

## 3. Discussion

In the present study, we investigated whether or not marliolide derivatives reduced melanin production in HRM-2 mice skin and further examined the underlying molecular mechanism regarding hypopigmentation effect of marliolide derivatives in association with autophagy. Marliolide and its derivatives showed significant melanin inhibition, and DMF02 was selected as one of the Nrf2 activators for the evaluation of hypopigmentation effect. A natural product, marliolide, has the structural features of γ-butyrolactone, and the aliphatic chain of 14 carbon length is connected to the α position of lactone ring by double bond with E-isomerism, and hydroxyl group is located at β position. DMF02 was designed based on the structure of marliolide, that is, hydroxyl group at β position was removed. UVB-induced hyperpigmentation in HRM-2 mice was reduced by topical application of DMF02. The loss of PMEL was observed in LC3-expressed areas of the mouse skin, indicating involvement of autophagy in the melanin suppressed areas. In in vitro experiments, DMF02 decreased melanin in B16F0 as well as in melanosomes-incorporated HaCaT cells by autophagy induction. These results were also verified in different melanocyte (MNT1 and HEM) and keratinocyte cell types (HEK) to raise reliability of our results. In addition, Nrf2 silencing by siRNA attenuated the DMF02-induced melanosome degradation via the decreased expression of p62 in melanocytes and keratinocytes. Collectively, all data indicated that marliolide derivatives induced melanosome degradation via Nrf2/p62-mediated autophagy.

Nrf2 protein is a 605 amino-acid transcription factor composed of 6 Nrf2-ECH homology (Neh) domains. Nrf2 facilitates the expression of various antioxidant genes [26,27] by binding to the antioxidant response element (ARE) binding site through the Neh domains. Nrf2 primarily protects the cell from oxidative stress by inducing antioxidant enzymes. Of note, Nrf2 negatively regulates melanogenesis by modulating PI3K/Akt signaling [23]. They also showed that overexpression of Nrf2 decreased the expression of TYRO and tyrosinase-related protein 1, and the inhibitory effect of Nrf2 was reversed by overexpression of Keap1. However, in our study, DMF02 induced melanin degradation without affecting melanogenic proteins. DMF02 did not affect both mRNA and protein levels of MITF and TYRO mRNA in vitro (Figure S5A–C). Wang et al. identified two marliolide derivatives from *Cinnamomum subavenium* with in vitro and in vivo screening systems by targeting the human TYRO [28]. They showed that two compounds (linderanolide B and subamolide A) exhibited mushroom TYRO inhibition. These two herbal compounds were proven to have inhibitory effects on melanin synthesis at low concentrations, and showed little cytotoxicity in normal human skin cells and zebrafish system. They proposed binding modes of linderanolide B in human TYRO, and two compounds have inhibitory effects on TYRO in a concentration-dependent manner. However, DMF02 used in this study induced melanin degradation via the autophagy mechanism without affecting TYRO.

Autophagy is a degradative mechanism that maintains cellular homeostasis by removing pathogens or dysfunctional components. Autophagy is known to play a role in melanosome biogenesis and melanosome degradation in the skin [10,29]. Nrf2 facilitates the transcription activity of p62 [30]; meanwhile, p62, as an autophagic cargo adapter, promotes autophagy [31,32]. Nrf2 reportedly binds to the ARE site in p62’s promoter region, thereby increasing p62 expression [30]. As a cargo protein for autophagosomes, p62 binds with polyubiquitinated proteins via the UBA domain, and self-oligomerizes through the PB1 domain [33]. Autophagic activity is increased by forming autophagosomes through the interaction between the LC3-II and LIR/LRs domains of p62 [34]. In the present study, we showed that Nrf2 activation promoted melanosome degradation by facilitating the expression of p62 and LC3-II (Figure S6). On the other hand, other groups examined the involvement of ATG7 in autophagy of melanocytes, where mice whose melanocytes or keratinocytes lacked Atg7 still retained functioning melanosome synthesis and transfer and displayed only moderate reduction of pigmentation [12]. 

Previous literatures reported the association between autophagy and melanin pigmentation. WD repeat domain phosphoinositide-interacting 1 and beclin 1 as potent autophagy regulators were involved in melanosome biogenesis [35,36]. Topical rapamycin improved hypopigmented macules in patients with tuberous sclerosis, who showed mTOR activation [37]. Autophagy inhibition in Unc-51 like kinase (ULK) knockdown increased melanogenesis in MNT-1 cells [38]. In addition, similarly to our study, autophagy-induced melanosome degradation was reported to regulate skin pigmentation [10,29]. Likewise, the role of autophagy in melanocytes showed conflicting results depending on the studies, cell types, and different signals in autophagy. However, these findings suggest that autophagy regulators could be applied for the regulation of pigmentation, even if they have pleiotropic or complicated roles in melanocyte biology [39]. 

An excessive accumulation of melanin in the skin results in hyperpigmentation disorders, such as melasma or freckles. In fact, tremendous efforts are undertaken to reduce melanin production in cosmetics and medicine. Most of the hypopigmentation agents were developed to target TYRO, such as commercially available hydroquinone and arbutin. However, tyrosinase inhibitors clinically have low efficacies and high side effects like irritation. Therefore, new target strategies for anti-melanogenic effects are needed, and autophagy regulators could be potentially promising options for skin rejuvenation and regulation of melanin pigmentation. In particular, as Nrf2 activators could also have an antioxidant activity in addition to p62-mediated autophagy induction, Nrf2 activator might be a promising way to develop anti-melanotic agents with a good safety profile.

In the present study, we showed that marliolide derivatives activated Nrf2 induced autophagy activity with melanosome degradation, therefore, they could be applied in skin-whitening agents after verifying their hypopigmentation effect and safety in humans through clinical studies.

## 4. Materials and Methods

### 4.1. Chemicals

Dihydro-5-Methylfuran-2(3H)-one derivatives (>99% purity) were synthesized as described in a previous study [24]. Pharmacological agents were dibutyryl-cAMP (Sigma-Aldrich, St. Louis, MO, USA) or forskolin (Sigma-Aldrich) as cAMP elevators; hydroquinone (Sigma-Aldrich) as a skin whitener; and CQ (Sigma-Aldrich) as a lysosomal inhibitor.

### 4.2. Cell Culture

B16F0 mouse melanoma cells (ATCC, Manassas, VA, USA) were cultured in DMEM (Sigma-Aldrich) supplemented with 10% FBS (Hyclone) and penicillin-streptomycin solution (Hyclone). Human epidermal melanocytes (HEM, Thermo Fisher Scientific, Waltham, MA, USA) were cultured in Medium 254 (Thermo Fisher Scientific) containing Human Melanocyte Growth Supplement (HMGS, Thermo Fisher Scientific). MNT1 human melanoma cells (ATCC) were cultured in MEM (Welgene, Korea) containing 10% DMEM, 20% FBS, and penicillin-streptomycin solution. HaCaT human keratinocytes (Thermo Fisher Scientific) were cultured in DMEM supplemented with 10% FBS and penicillin-streptomycin. Human epidermal keratinocytes (HEK, Thermo Fisher Scientific) were cultured in Medium 254 (Thermo Fisher Scientific) containing Human Melanocyte Growth Supplement (HMGS, Thermo Fisher Scientific). These cells were incubated at 37 °C with 5% CO_2_.

### 4.3. Melanin Quantification

Cells were treated for 72–96 h with α-MSH (Sigma-Aldrich). Extracellular melanin and intracellular melanin were homogenized in 0.85 N NaOH and 20% dimethyl sulfoxide (Sigma-Aldrich) at 80 °C, and absorbance values were measured at 405 nm.

### 4.4. UVB-Induced Skin Hyperpigmentation

Six-week-old male HRM-2 mice (Central Lab Animals, Seoul, Korea) were maintained at 22 ± 2 °C on a 12 h/12 h light/dark cycle with a humidity of 55% ± 5% and ad libitum access to water and food. The dorsal skin of each mice was divided into three 2 cm × 1.5 cm parts. The mice were exposed to UVB (150–250 mJ/cm^2^) radiation for four consecutive weeks to induce skin pigmentation.

0.1% DMF02 was dissolved in a vehicle (propylene glycol:ethanol = 8:2) or 2% of hydroquinone. The vehicle solutions, 0.1% DMF02 or 2% HQ, were topically applied to an allocated skin area on the mice’s dorsal side once-daily for 21 consecutive days. Skin pigmentation was measured every 3 days after the end of UVB radiation by chromameter. Skin tissues were fixed in 10% formaldehyde (Sigma-Aldrich), sectioned into 5-μm sections, and stained with Fontana-Masson silver nitrate (Scytek) or underwent immunohistochemistry staining. Protein extracts were prepared from fresh tissues and subjected to western blot analysis. 

The protocols were conducted in accordance with the Korean Ministry of Food and Drug Safety Guide for the Care and Use of Laboratory Animals and approved by the Animal Experimentation Ethics Committee in Yonsei National University (permit number IACUC-A-202001-1005-01, 29 January 2020).

### 4.5. Isolation of Melanosomes and Incubation of Isolated Melanosomes in HaCaT Cells

Melanosome isolation was performed according to the previous method with slight modifications [26]. In short, MNT-1 cells were homogenized in 1 mL of ice-cold lysis buffer (20 mM Tris-HCl (pH 7.2), 150 mM NaCl, 1 mM EDTA, and 250 mM sucrose). The solution was then mixed three times for 30 min at 4 °C. The nuclei and cell debris were removed after centrifugation at 1000× *g* for 5 min at 4 °C. The supernatant was layered on a sucrose gradient by centrifugation at 20,000× *g* for 30 min at 4 °C. The pellet was gently washed three times in PBS. Isolated melanosomes were incubated in culture media of HaCaT cells for 3 days until sufficient numbers of melanosomes were taken into HaCaT cells. Then, the culture media of HaCaT cells were washed with PBS three times to get rid of melanosomes, which were not transferred into HaCaT cells. DMF01 was treated into melanosome-incorporated HaCaT cells for 3 days to see the change in melanosomes taken within HaCaT cells. 

### 4.6. Transfection and Plasmids

The mRFP-eGFP-LC3 construct was kindly provided by Dr. Tamotsu Yoshimori (Osaka University, Osaka, Japan). Cells were seeded into a chamber slide and cultured overnight. B16F0 melanoma cells or HaCaT cells were transfected with ptfLC3-expressing plasmid using TransIT-2020 transfection reagent according to the manufacturer’s protocol (Mirus, Madison, WI, USA) for 24 h and then treated with DMF02 for 24 h. The cells were then washed in PBS and fixed in freshly prepared 4% paraformaldehyde. After three washes in PBS, the cells were mounted with 4’, 6-diamidino-2-phenylindole (DAPI). Autophagic flux was determined based on patterns of green fluorescent protein (GFP) and red fluorescent protein (RFP) puncta under a Zeiss confocal lase-scanning microscope. 

### 4.7. Western Blot Analysis

Proteins were isolated from cells using RIPA buffer (iNtRON, korea), separated by 10% SDS-PAGE, and transferred to polyvinylidene fluoride (PVDF) membranes (Millipore). The blots were incubated with 5% non-fat milk (Becton-Dickinson) in Tris-buffered saline containing 0.05% Tween 20 (TBST) at room temperature for 1 h. After washing three times with TBST, the blots were incubated with primary antibodies overnight at 4 °C and then with secondary antibody for 1 h at room temperature. Bound antibody was detected with an enhanced chemiluminescence reagent (GE Healthcare). Primary antibodies were TYRO (Santa Cruz Biotechnology), MITF-M (Abcam), Nrf2 (Cell Signaling Technology), p62 (Cell Signaling Technology), ATG5-ATG12 complex (Cell Signaling Technology), PMEL (Abcam), LC3A/B (Cell Signaling Technology), or β-actin (Santa Cruz Biotechnology). Secondary antibodies were rabbit anti-goat IgG labeled with horseradish peroxidase (HRP) (Thermo Fisher Scientific), goat anti-rabbit IgG labeled with HRP (Thermo Fisher Scientific), and goat anti-mouse IgG labeled with HRP (Thermo Fisher Scientific).

### 4.8. Real-Time PCR Analysis

Total cellular RNA was isolated using NucleoZOL (Macherey-Nagel) according to the manufacturer’s instructions. cDNAs were synthesized using a premix reagent kit (iNtRON, Seongnam-Si, Korea). The real-time PCR was performed using Applied Biosystems’ PCR machine (Applied Bioscience, StepOne). The primer sequences used were as follows: mouse p62 sense, 5′-ATGTGGAACATGGAGGGAAGA-3′, and antisense, 5′-GGAGTTCACCTGTAGATGGGT-3′; human p62 sense, 5′-GACTACGACTTGTGTAGCGTC-3′, and antisense, 5′-AGTGTCCGTGTTTCACCTTCC-3′; mouse MITF sense, 5′-AGGACCTTGAAAACCGACAG-3′, and antisense, 5′-GTGGATGGGATAAGGGAAAG-3′; mouse TYRO sense, 5′- AGCCTGTGCCTCCTCTAA -3′, and antisense, 5′- AGGAACCTCTGCCTGAAA-3′, with mouse GAPDH sense, 5′-TGACCTCAACTACATGGTCTACA-3′, and antisense, 5′-CTTCCCATTCTCGGCCTTG-3′; human GAPDH sense, 5′- GTCTCCTCTGACTTCAACAGCG-3′, and antisense, 5′- ACCACCCTGTTGCTGTAGCCAA -3′.

### 4.9. Small Interfering RNA Knockdown

Cells were transfected with Nrf2 or p62 siRNA or ATG5 siRNA with Lipofectamine (Thermo Fisher). The siRNAs of Nrf2 #1 (5′-UUGGGAUUCACGCAUAGGAGCACUG-3′), Nrf2 #2 (5′-CCGAAUUACAGUGUCUUAAUU-3′), p62 #1 (5′-CUUGUAGUUGCAUCACGUA-3′), p62 #2 (5′-CCGCAUCUACAUUAAAGAGAA-3′), ATG5 #1 (5′-CUGUCUUUGCUGUUACGUU-3′), and ATG5 #2 (5′-AACGUAACAGCAAAGAGAG-3′) were constructed. 

### 4.10. Statistical Analysis

Data were analyzed with ANOVA followed by the Student’s *t*-test and demonstrated as the mean ± standard error of the mean (SEM). *p* values < 0.05 were considered statistically significant.

## Data Availability

Data sets related to this article can be found at https://data.mendeley.com/datasets/fpcx2rfcb6/1 (accessed on 13 April 2021), an open-source online data repository hosted at Mendeley Data (Yun et al., 2020).

## Supplementary Materials

The following are available online at https://www.mdpi.com/article/10.3390/ijms22083995/s1.
