# Supplementary material for: Marliolide Derivative Induces Melanosome Degradation via Nrf2/p62-Mediated Autophagy
Source: Int J Mol Sci. 2021 Apr 13;22(8):3995. doi: 10.3390/ijms22083995 (PMC8070456; doi:10.3390/ijms22083995)
Supplement: Supplementary file 1 [file ijms-22-03995-s001.pdf]

Supplementary Material

Marliolide derivative induces melanosome degradation via Nrf2/p62-mediated autophagy

Cheong-Yong Yun[1], Nahyun Choi[1], Jae Un Lee[2], Eun Jung Lee[3,4], Ji Young Kim[3], Won Jun Choi[2], Sang Ho Oh[3,*], and Jong-Hyuk Sung[1,5,*]

[1] STEMORE Co. Ltd., Incheon, South Korea; cyyun@stemore.co.kr (C.Y.Y.); nh147837@gmail.com (N.H.C.); brian99@yonsei.ac.kr (J.H.S)

[2] College of Pharmacy, Dongguk University-Seoul, Goyang, Gyeonggi-do, Korea; ljwoon55@naver.com (J.U.L); mp89@dongguk.edu (W.J.C)

[3] Department of Dermatology and Cutaneous Biology Research Institute, Severance Hospital, Yonsei University College of Medicine, Seoul, Korea; leeej87@yuhs.ac (E.J.L); snyd@yuhs.ac (J.Y.K); oddung93@yuhs.ac (S.H.O)

[4] Brain Korea 21 PLUS Project for Medical Science, Yonsei University College of Medicine, Seoul, Korea; ; leeej87@yuhs.ac (E.J.L)

[5] College of Pharmacy, Institute of Pharmaceutical Sciences, Yonsei University, Incheon, Korea; brian99@yonsei.ac.kr (J.H.S)

* Correspondence: brian99@yonsei.ac.kr; Tel.: +82-32-749-4506; oddung93@yuhs.ac; Tel.: +82-2-2228-2080

Contents

◦ Figures S1 to S6

Figure S1

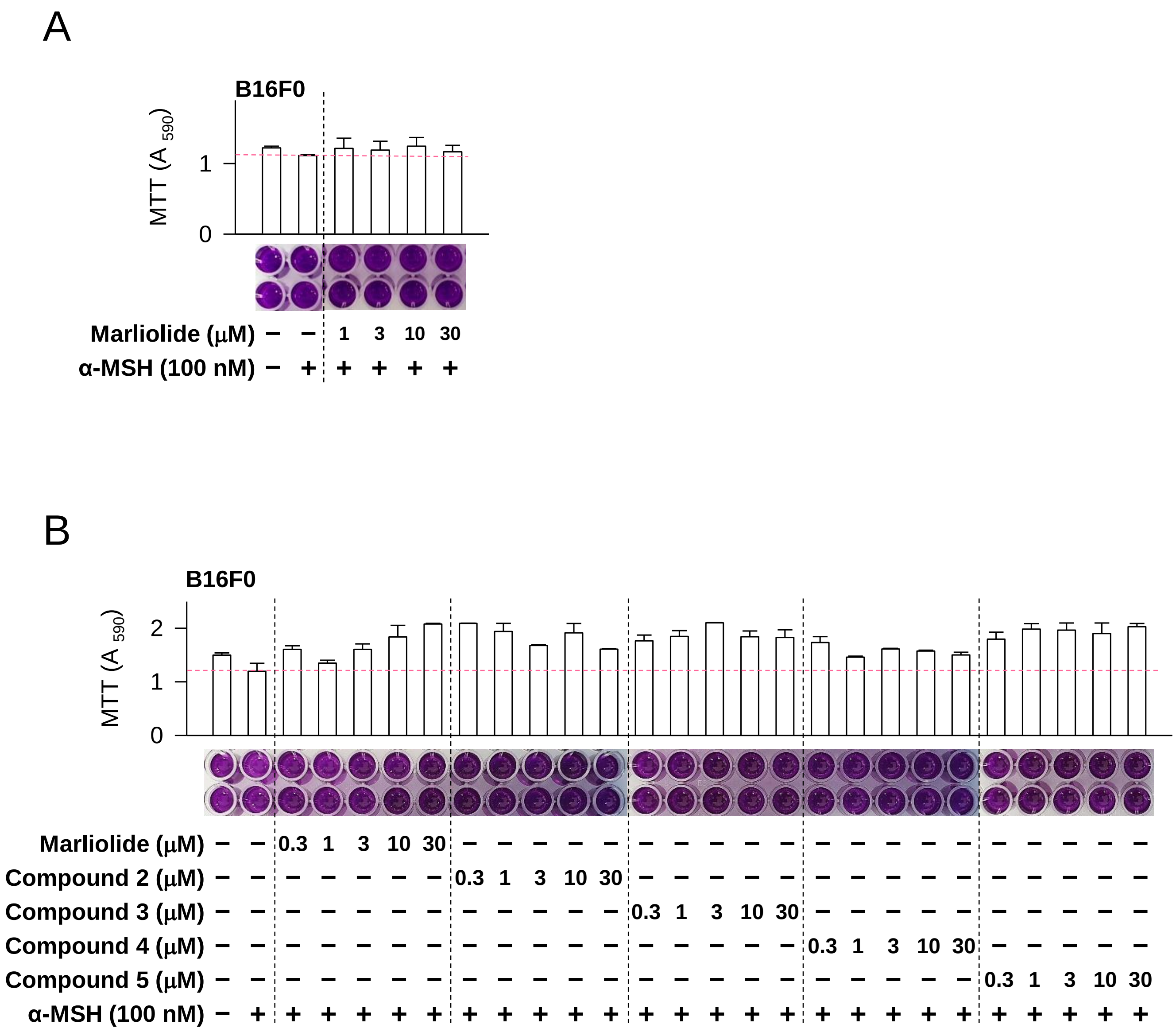

Figure S1. Effect of Marliolide or Marliolide derivatives on cell viability. B16F0 melanoma cells were stimulated with α-MSH for 72 h in the presence of Marliolide (A) or Marliolide derivatives (B). MTT (50 μg/ml) were added to each well and incubated at room temperature for 30 min. Formazan crystals were dissolved in DMSO and measured absorbance values at 590 nm. All values are expressed as mean ± SEM from two independent experiments in duplicate.

Figure S2

A

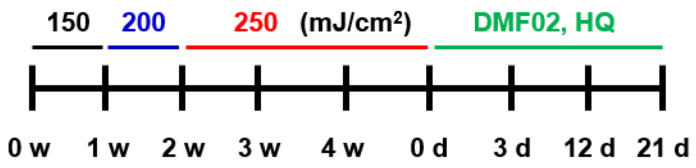

Figure S2. Experimental design. (A) The dorsal skin of HRM2 mice were exposed to UVB (150–250 mJ/cm[2]) four weeks, and topically applied with 0.1 % DMF02 or 2 % HQ in a once-daily regimen for twenty-one days.

Figure S3

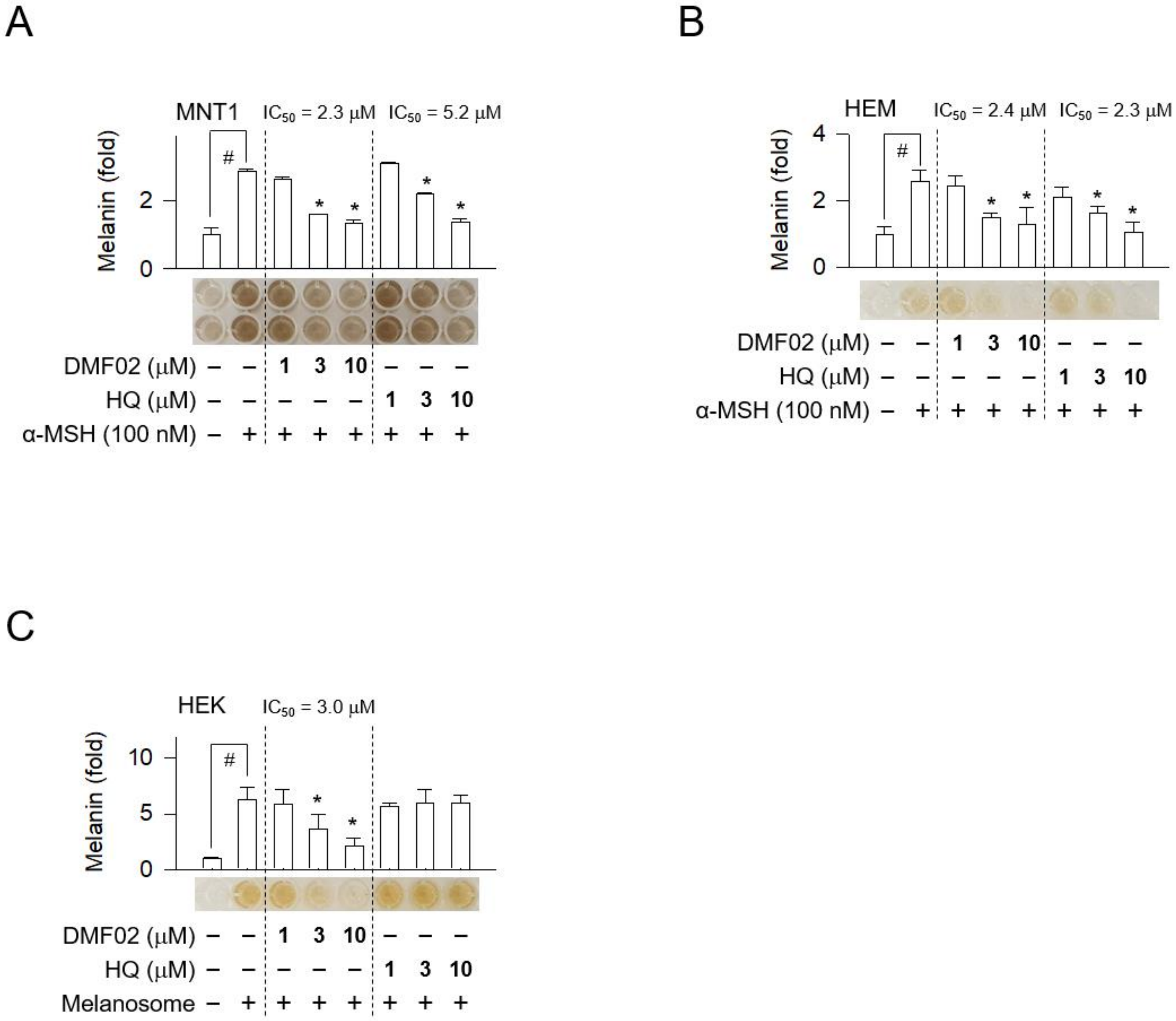

Figure S3. Effect of DMF02 on melanin production or transferred melanosomes. (A) MNT1 cells were stimulated with α-MSH for 72 h in the presence of DMF02. (B) HEM cells were stimulated with α-MSH for 96 h in the presence of DMF2. (C) HEK cells were treated with isolated melanosomes for 72 h in the presence of DMF02. The amount of melanin was quantified by absorbance values were measured at 405 nm and are represented as a relative fold. All values are expressed as mean ± SEM from two independent experiments in duplicate. #P < 0.05 vs. medium alone-added group. *P < 0.05 vs. α-MSH or melanosome-treated group. The difference was determined using the Student's t-test.

Figure S4

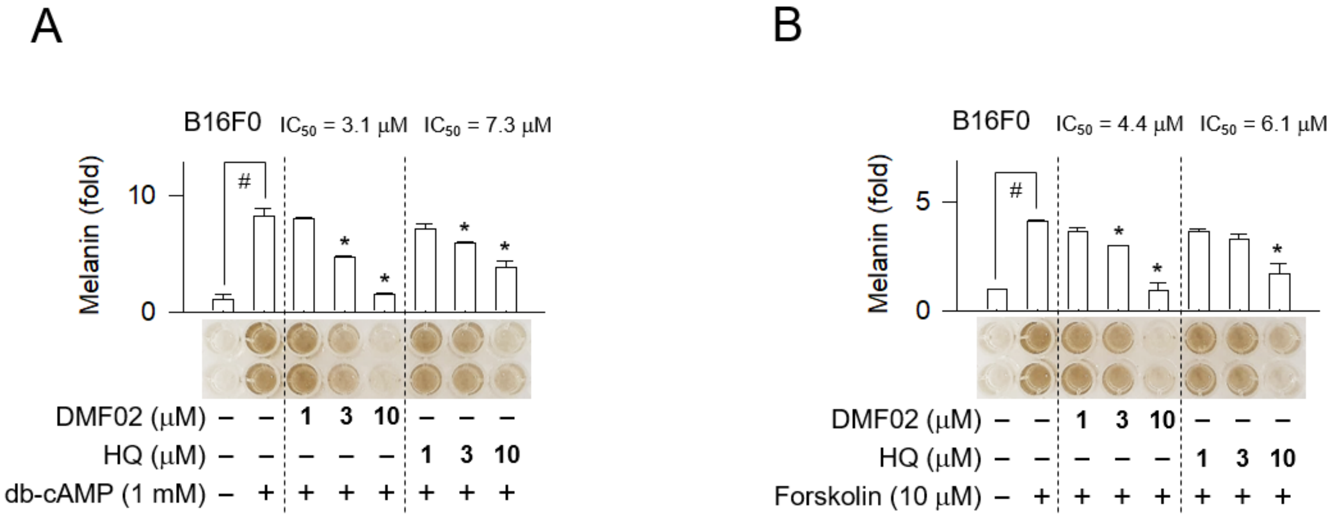

Figure S4. Effect of DMF02 on melanin production in B16F0 melanoma cells stimulated with other cAMP elevators. B16F0 melanoma cells were stimulated with db-cAMP (A) or forskolin (B) for 72 h in the presence of DMF02 or HQ. The amount of melanin was quantified by absorbance values were measured at 405 nm and are represented as a relative fold. All values are expressed as mean ± SEM from two independent experiments in duplicate. #$P < 0.05$ vs. medium alone-added group. *$P < 0.05$ vs. db-cAMP or forskolin-treated group. The difference was determined using the Student's t-test.

Figure S5

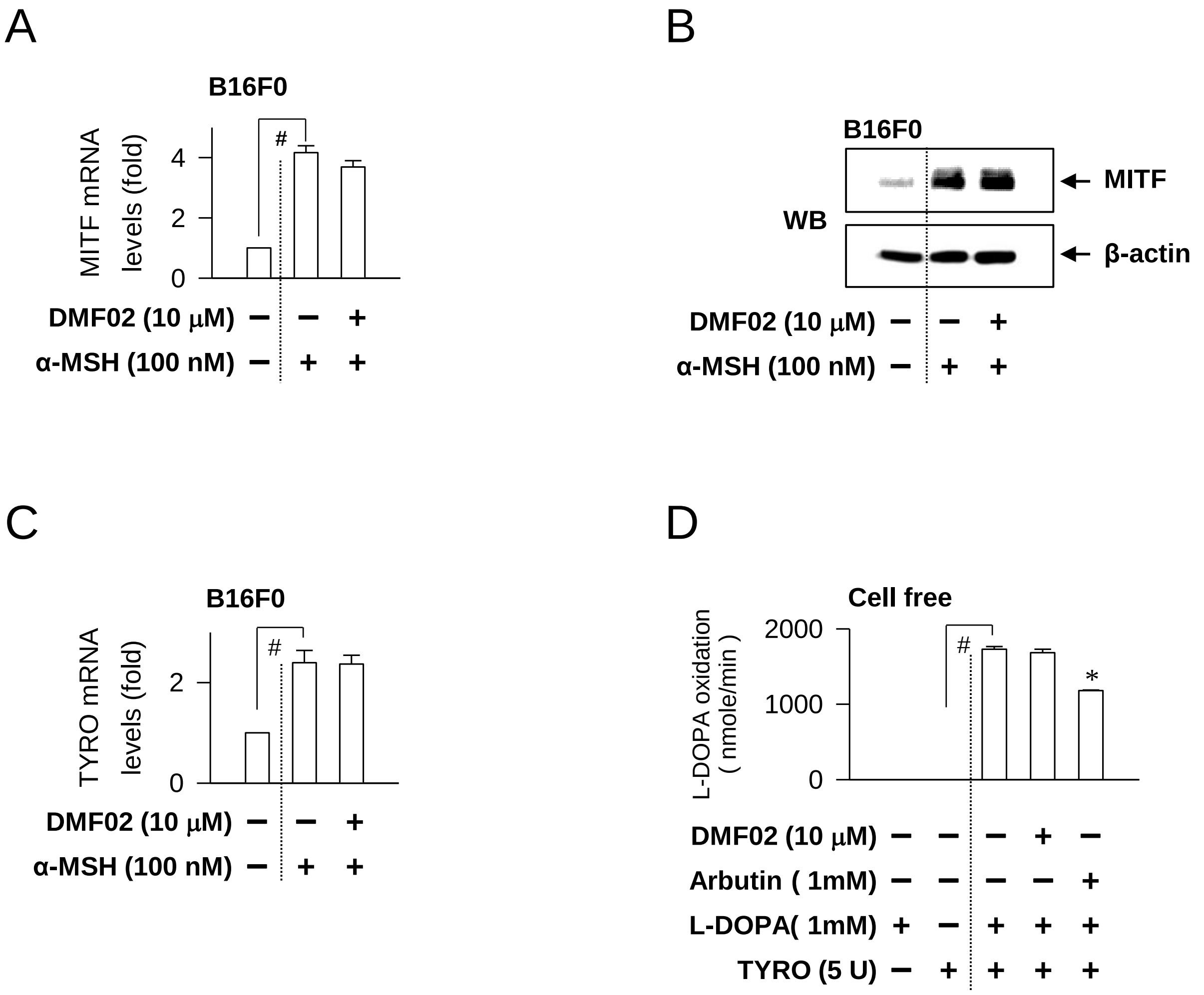

Figure S5. Effect of DMF02 on major melanogenic proteins. Real-time PCR on the induction of MITF or TYRO. B16F0 melanoma cells were stimulated with α-MSH for 2 h (A) or 18 h (C) in the presence of DMF02. (B) WB of MITF. B16F0 melanoma cells were stimulated with α-MSH for 4 h in the presence of DMF02. (D) Effects of DMF02 on the TYRO activities. TYRO activity was determined in the presence of L-DOPA, as a substrate, and are represented as nmole/min. Data are mean ± SEM. #P < 0.05 vs. medium alone-added group. *P < 0.05 vs. L-DOPA and TYRO-treated group. The difference was determined using the Student's t-test.

Figure S6

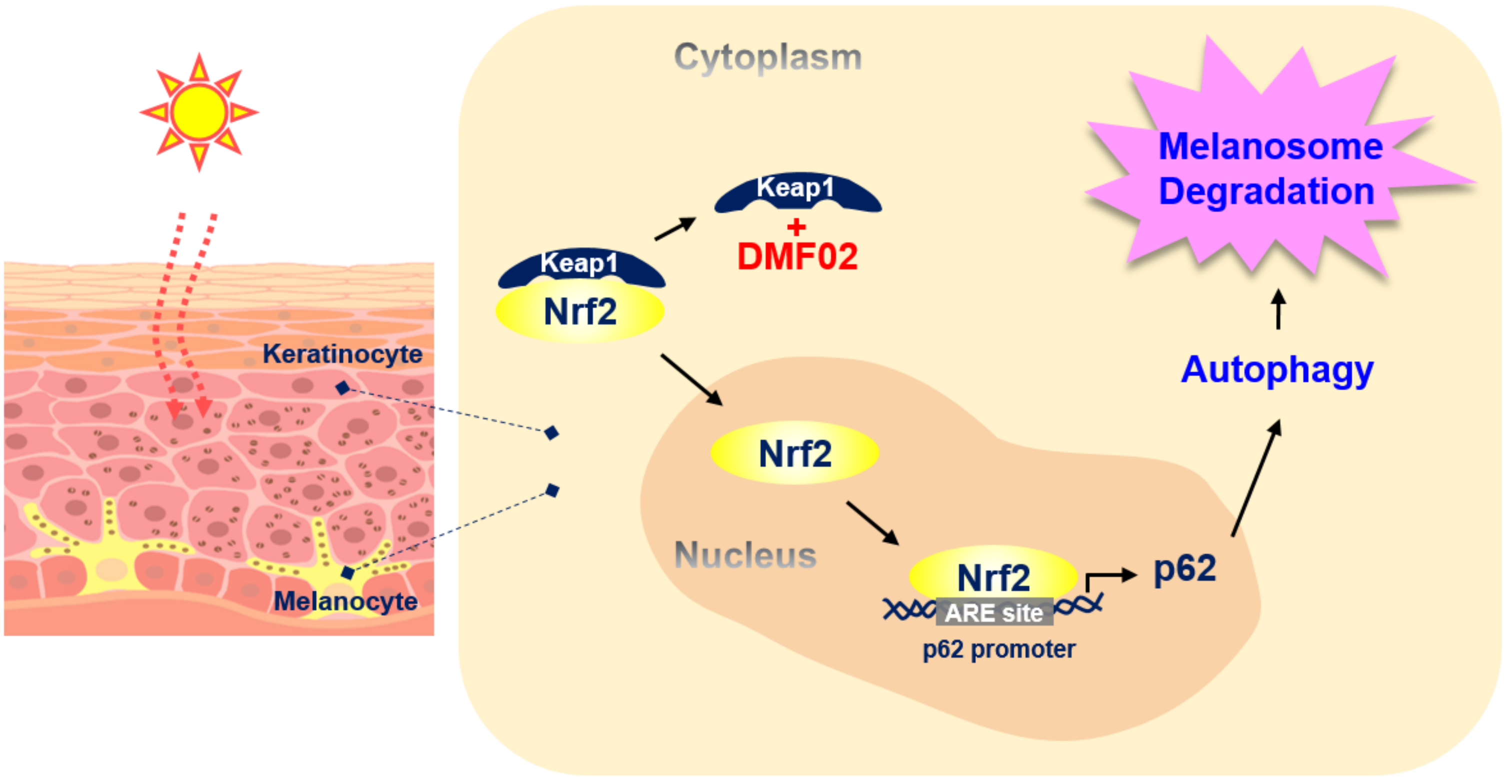

Figure S6. A suggested mechanism of DMF02 on melanosome reduction. DMF02 binds to keap1 to activate Nrf2. Nrf2 translocates to the nucleus and binds to the ARE site located at the promoter region of p62. p62 activates the autophagy pathway and increased autophagy led to melanosome degradation in melanocytes and keratinocytes. Taken together, DMF02 induced the reduction of melanosome in keratinocytes and melanocytes via the Nrf2/p62-mediated autophagy activation.
